# Angulin-2/ILDR1, a tricellular tight junction protein, does not affect water transport in the mouse large intestine

**DOI:** 10.1038/s41598-020-67319-5

**Published:** 2020-06-25

**Authors:** Wendy Hempstock, Shiori Sugioka, Noriko Ishizuka, Taichi Sugawara, Mikio Furuse, Hisayoshi Hayashi

**Affiliations:** 1 Laboratory of Physiology, Graduate School of Nutritional and Environmental Sciences, University of Shizuoka, 52-1 Yada, Suruga-ku, Shizuoka, 422-8526 Japan; 2 Department of Histology, Graduate School of Medical Sciences, Kumamoto University, 1-1-1 Honjo, Chuo-ku, Kumamoto, 860-8556 Japan; 3 Department of Cellular Structure, National Institute for Physiological Sciences, 5-1 Higashiyama Myodaiji, Okazaki, 444-8787 Japan

**Keywords:** Physiology, Gastroenterology

## Abstract

Angulin-2/ILDR1 is a member of the angulin protein family, which is exclusively expressed at tricellular tight junctions in epithelia. Tricellular tight junctions are found where three cells meet and where three bicellular tight junction strands converge. Tricellular tight junctions are thought to be important for paracellular permeability of ions and water in epithelial tissues. It was recently reported that angulin-2/ILDR1 knockout mice have water transport abnormalities in the kidney. Since angulin-2/ILDR1 is the main tricellular tight junction protein in the large intestine, the goal of this research was to examine the effect of angulin-2/ILDR1 knockout on large intestinal paracellular water transport. We found that *Ildr1* knockout mice showed no detectable phenotype other than deafness. In addition, paracellular transport as assessed by Ussing chamber was unchanged in *Ildr1* knockout mice. However, we found that in the colon and the kidney of *Ildr1* knockout mice, another tricellular tight junction protein, angulin-1/LSR, changes its expression pattern. We propose that with this replacement in tissue localization, angulin-1/LSR compensates for the loss of angulin-2/ILDR1 and maintains the barrier and function of the epithelia in the large intestine as well as the kidney.

## Introduction

Water is essential for all living things. In the intestinal tract, more than 10 L of water is (re)absorbed per day in humans. It is estimated that the colon absorbs about 90% of the water that enters the large intestine^1,2^. A fundamental question in gastrointestinal physiology is how the intestine transports water. It has been shown that intestinal solute transport is coupled to water transport and the osmolality of transported fluid in the intestine increases in a proximal to distal fashion^3^. In the large intestine, water is transported against osmotic gradients across the intestinal epithelia as in the kidney^4^. However, water absorption mechanisms in the colon are still a matter of debate^5^.

The pathway of water transport in the intestinal epithelium is thought to occur via transcellular and paracellular routes. Transcellular water transport occurs via aquaporins^6–8^. Aquaporins are important for water transport in epithelial tissues with high fluid transport rates, such as the kidney or salivary glands. In the intestine, however, the functional significance of aquaporins has not been established^8–11^, since aquaporin KO (AQP3, 4 and 8) mice did not manifest in severe water transport impairment in the large intestine^9–11^. Water transport is also thought to occur via paracellular transport^12,13^. However, the quantitative contribution of each pathway to total fluid transport in the intestine still remains to be established.

It has been proposed that water transport in leaky epithelial tissues, like the small intestine, occurs isotonically: in the absence of an osmotic difference between the luminal compartment (around 300 mOsm/kg H_2_O) and the blood, and coupled with active transport of solutes^14^. The mechanism of water transport is explained by the standing gradient theory, which assumes the lateral intracellular spaces between cells to be cylindrical tubes, with a closed and open end, where active transport of solute occurs near the closed end (tight junctions) and osmotic transport of water follows, which creates an osmotic gradient along the length of the channel^14^. This theory explains how water can be absorbed isotonically from the small intestinal lumen to blood. However, in the large intestine, the osmolality of the luminal contents is hypertonic (approximately 500 mOsm/kg H_2_O)^3^, so water absorption must occur against a greater osmolality gradient^4^. Therefore, it is thought that an osmotic barrier is needed to keep this osmotic gradient between the luminal compartment and the blood^5^. Paracellular transport involves specialized structures called tight junctions (TJ) that determine the barrier and permselectivity of the paracellular pathway^15^. TJ are located at the borders where two or more cells meet. Bicellular TJ (bTJ) are composed of proteins called claudins, which are important for the barrier and permselectivity^16^. To date, 27 members of the claudin family have been identified^16^. Claudins 2 and 15 are channel forming claudins, which act as pores for solutes like Na^+^, and in theory transport water as well^17–19^. In the large intestine, bTJ are not thought to be the main route of paracellular water transport and it is theorized that tricellular TJ (tTJ) instead are important for water absorption^18^.

Tricellular TJ are junctions where three cells meet and they require a specialized structure. In the center of tTJ there is a central sealing element formed by the attachment of sealing elements from each of the three cells which come together to form a tube of about 1 µm long and 10 nm across^20^. Angulin proteins form the central sealing element. In the large intestine, angulin 2 (Immunoglobulin-like domain containing receptor 1; ILDR1) is the main tTJ protein^21^. The other angulin proteins are lipolysis stimulated receptor (LSR/angulin 1), immunoglobulin-like domain containing receptor 2 (ILDR2; angulin 3), and they along with ILDR1 are required to recruit another tTJ protein tricellulin to the tTJ^21^. Tricellulin was recently found to play a role in paracellular water transport in MDCK c7 cells^22^.

It was shown that KO of ILDR1 in mice results in abnormal water transport in the kidney^23^. ILDR1 KO mice had impaired urine concentration and it was determined that transport of water rather than ions was impacted by the loss of angulin 2/ILDR1^23^. Since angulin 2/ILDR1 KO mice have been shown to have disrupted water transport in the kidney and angulin 2/ILDR1 is the main tTJ protein in the large intestine, the goal of this research was to study the effect of angulin 2/ILDR1 KO on paracellular water transport in the large intestine.

## Results

### Investigation of metabolic changes and residual angulin-2/ILDR1 protein expression in the colon of Ildr1 KO mice

**Figure 1:**
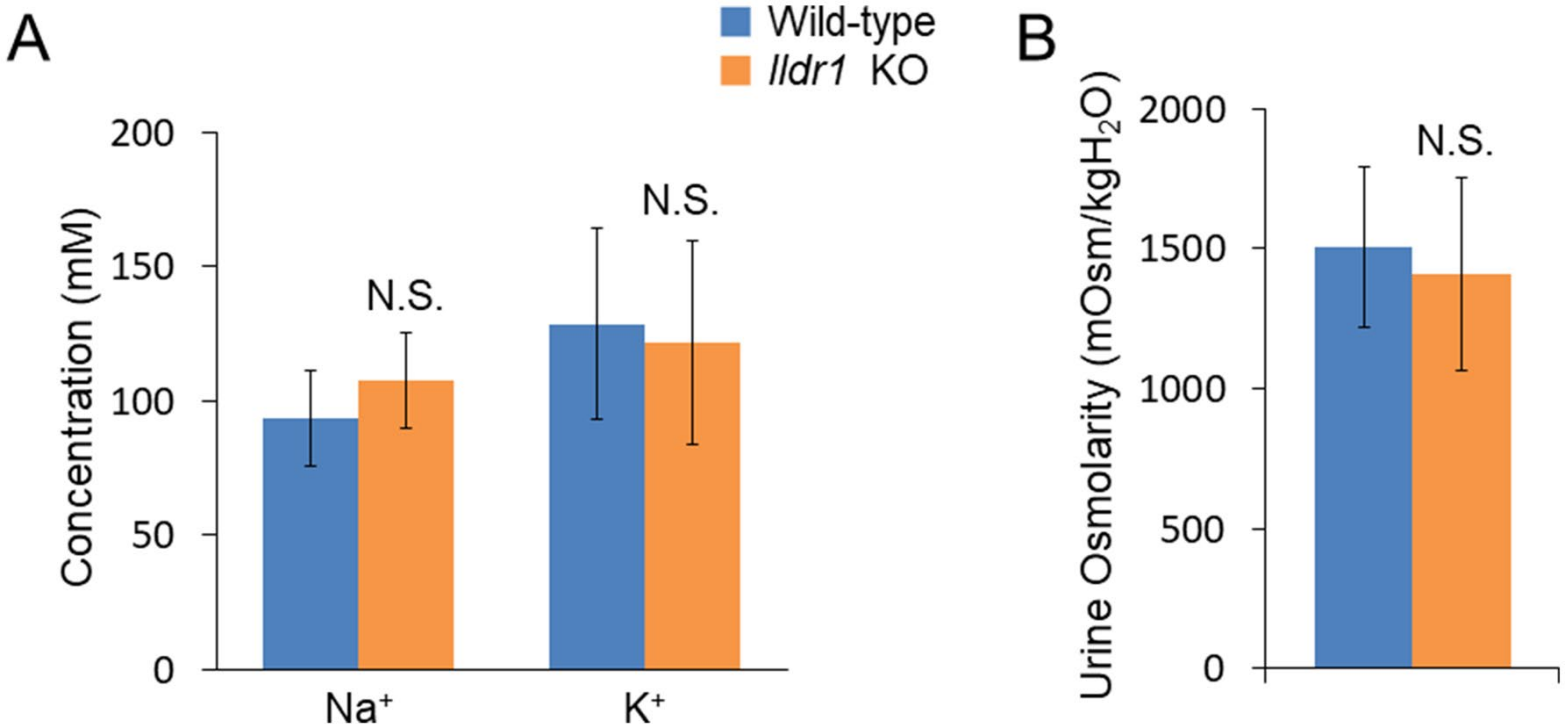
Fresh urine analysis in *Ildr1* KO and WT mice. Fresh urine was collected directly from the bladder and osmolality was measured using an osmometer. Na^+^ and K^+^ were measured using ion electrodes. (**A**) Na^+^ and K^+^ concentration of fresh urine from *Ildr1* KO and WT mice, n = 4 and 6, KO and WT respectively. (**B**) Osmolality of fresh urine from *Ildr1* KO and WT mice, n = 4 and 7, KO and WT respectively. *N.S.* not significant compared with wild-type mice.

**Figure 2:**
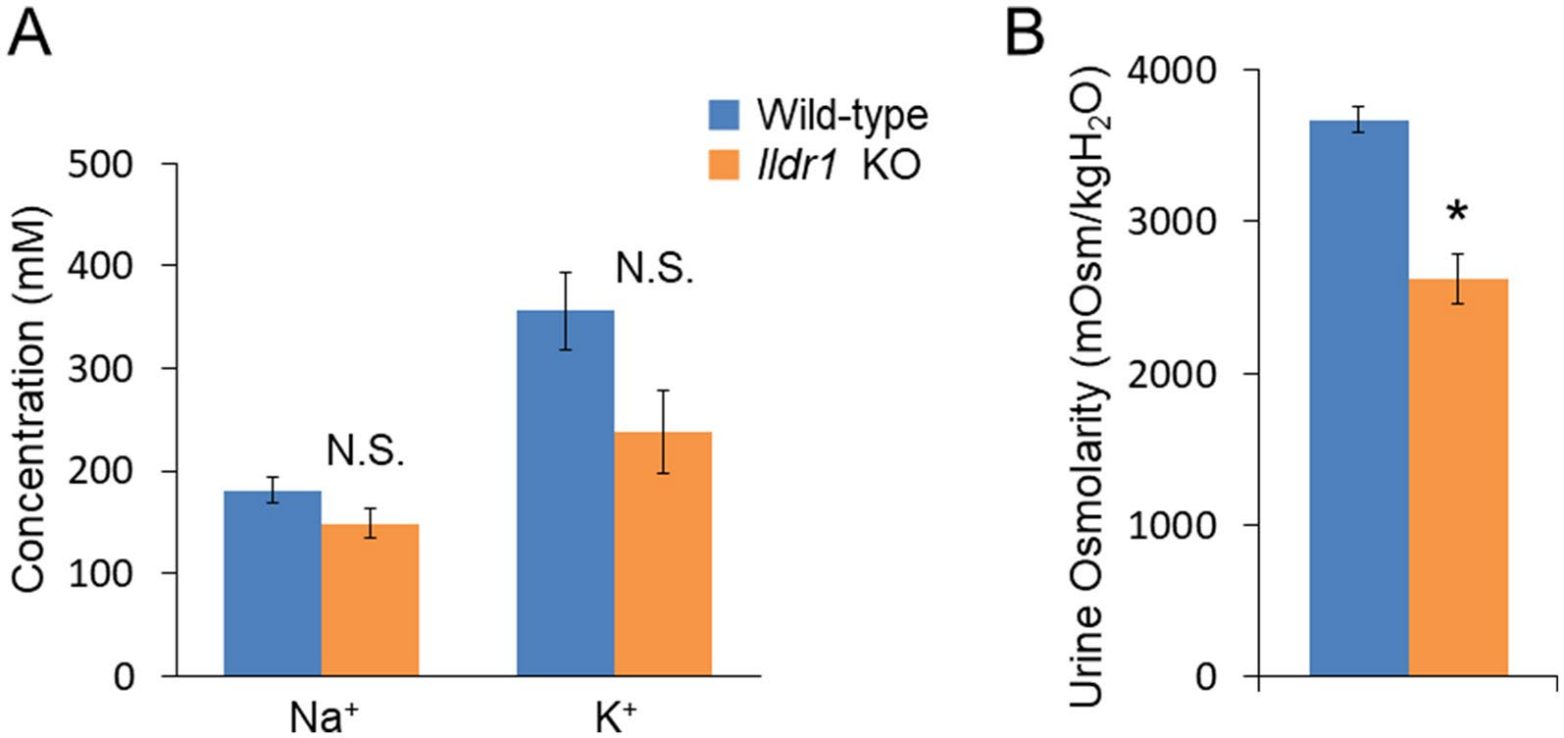
Fresh urine analysis in *Ildr1* KO and WT mice under water-restricted conditions. (**A**) Na^+^ and K^+^ concentration of fresh urine from water-restricted *Ildr1* KO and WT mice, n = 4 and 6, KO and WT respectively. (**B**) Osmolality of fresh urine from water restricted *Ildr1* KO and WT mice, n = 4 and 7, KO and WT respectively. *p < 0.05 as compared with wild-type mice. *N.S.* not significant compared with wild-type mice.

**Figure 3:**
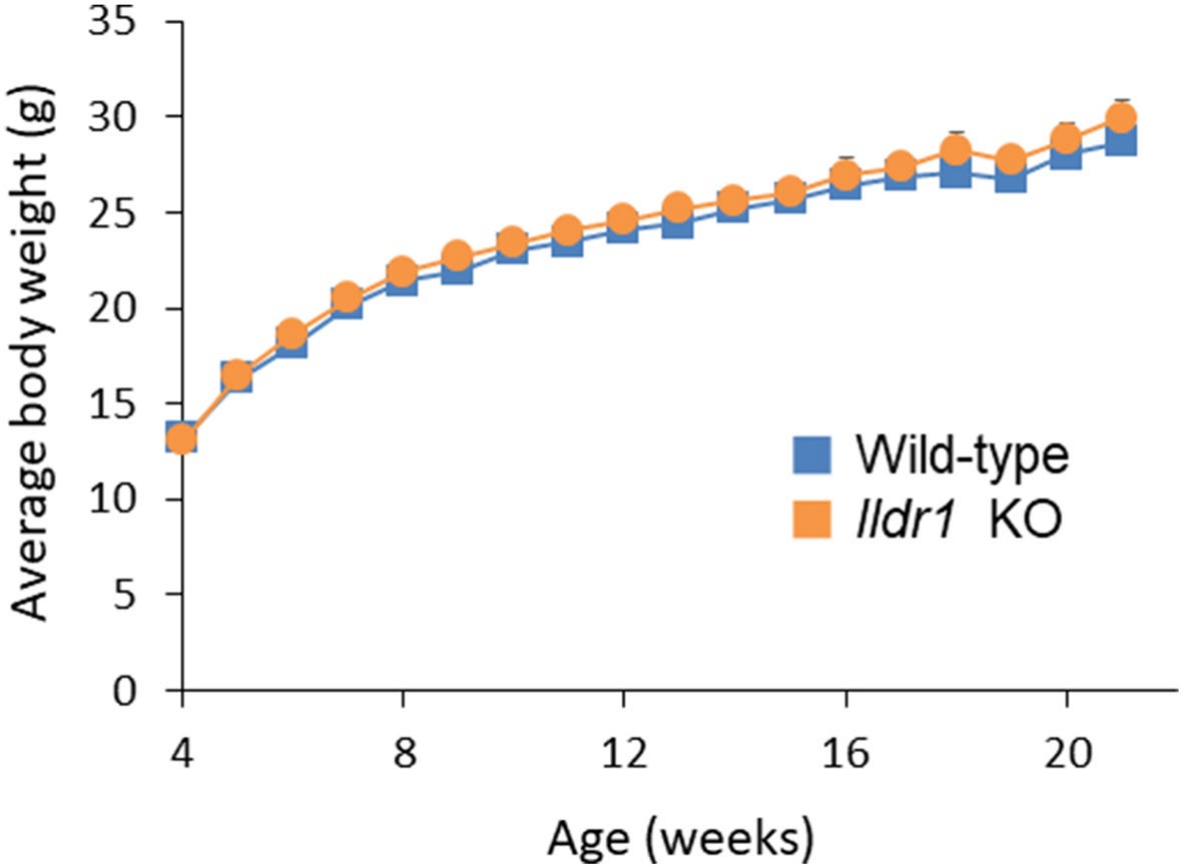
Growth curves of *Ildr1* KO and WT mice. Mice were weighed weekly starting from weaning until 21 weeks of age. The values represent the mean body weight, with the error bars representing SEM, n = 4–15 and 10–18 for WT and KO, respectively.

**Figure 4:**
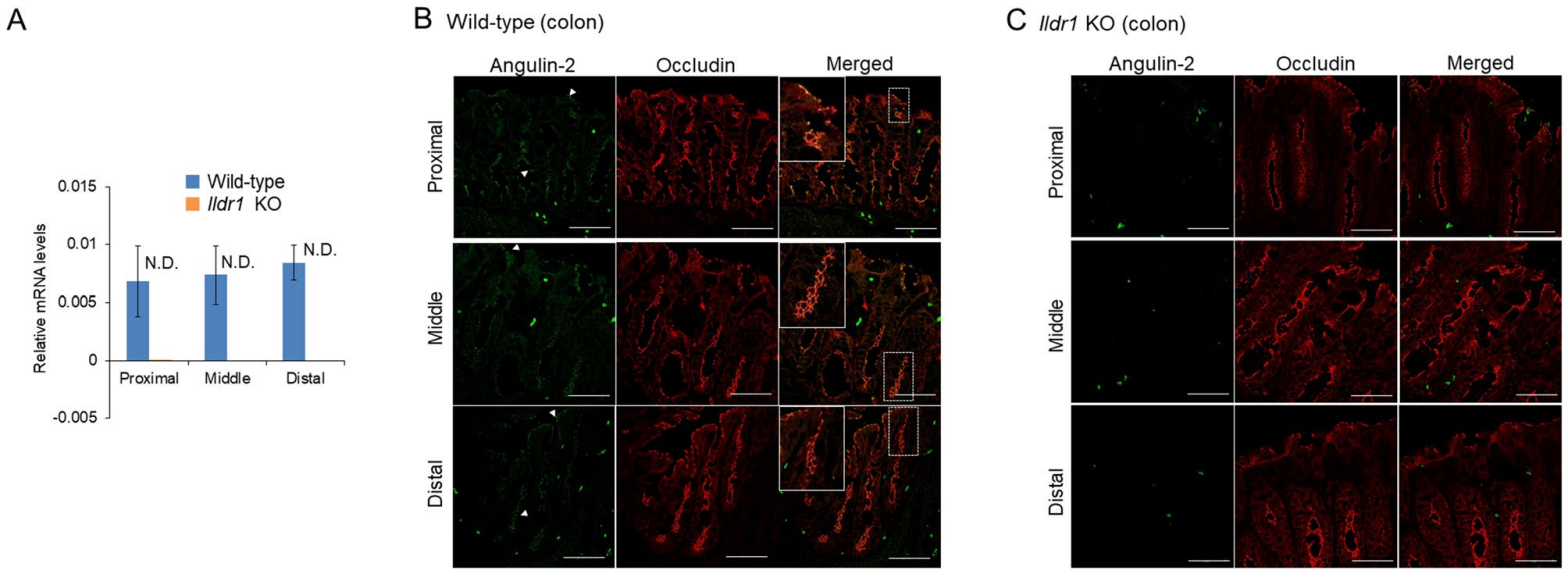
Confirmation of *Ildr1* KO in mice. *Ildr1* KO was confirmed by real-time quantitative PCR and immunofluorescence. (**A**) Relative mRNA levels of ILDR1. The values represent the mean, n = 5. Error bars represent the SEM. *N.D.* not detected. (**B**) Representative immunofluorescence images of WT mouse proximal, middle, and distal large intestine stained with occludin (red) and angulin 2/ILDR1 (green). The dashed line rectangles in the merged images indicate the focus area, which are enlarged in the solid rectangles. (**C**) Representative images of *Ildr1* KO mouse proximal, middle, and distal large intestine stained with occludin (red) and angulin 2/ILDR1 (green). For immunofluorescence, scale bar represents 50 µm.

It has been shown that growth retardation, polydipsia and polyuria were manifested in *Ildr1* KO mice due to a defect of urine concentrating mechanisms^23^. We first ascertained the impact of deficiency of angulin-2/ILDR1 on the macroscopic phenotype by performing metabolic cage experiments using *Ildr1* KO mice and comparing with wild-type (WT) mice. The results of the metabolic cage experiments are shown in Table 1. There was no difference in 24 h urine (p = 0.57) and stool output (p = 0.69), water and food intake (p = 0.10 and p = 0.85, respectively), or fresh fecal water percentage (p = 0.64). Since it was previously observed that *Ildr1* KO mice cannot concentrate urine^23^, fresh urine was collected directly from the bladder of *Ildr1* KO and WT mice and Na^+^ and K^+^ concentration as well as osmolality were measured (Fig. 1). There was no difference in fresh urine ion concentration (Fig. 1A; p = 0.59 and p = 0.90, for Na^+^ and K^+^, respectively), or osmolality (p = 0.83). In a dehydration challenge, Na^+^ and K^+^ concentration and osmolality were measured after 24 h of water restriction (Fig. 2). *Ildr1* KO mice tended to have lower K^+^ concentration (Fig. 2A, p = 0.08) and were able to concentrate urine after 24 h water restriction, but to a lesser degree than WT mice (Fig. 2B, p < 0.003). These results suggest that the phenotype that was previously shown in *Ildr1* KO mice^23^ may be a late-onset phenotype or that angulin-2/ILDR1 protein was not fully deleted in our *Ildr1* KO mice. To assess the first possibility, we monitored the growth of male *Ildr1* KO mice for 21 weeks and constructed a growth curve (Fig. 3). Taking the average body weight of all male *Ildr1* KO mice and WT mice resulted in no difference between the animals. Furthermore, a limited number of metabolic cage experiments were performed using older mice (5 months of age). However, there were no discernable differences between older *Ildr1* KO mice and young WT mice (Table 1, third column). We next considered the second possibility that angulin-2/ILDR1 was not completely deleted in our *Ildr1* KO mice. To date, two different null mutant alleles of mouse *Ildr1* have been used to characterize the impact of loss of angulin-2/ILDR1 in mice. One is constructed with a gene trap in intron 2^24^, which was the same null mutant allele used in the renal experiments^23^, and the other has exons 3–5 deleted^25^, which was the model used in this study. Interestingly, both *Ildr1* KO mouse models manifested in deafness, suggesting that the models are not essentially different from each other^25,26^. However, it has been shown that human ILDR1β, which lacks a transmembrane-spanning domain that is encoded by exon 5, is expressed in the cytosol and not in plasma membrane^27^. This study implies that the residual cytosol domain of angulin-2/ILDR1 could still be expressed in the cytosol. To assess this possibility, real time qPCR using primers for angulin-2/ILDR1 was performed for each segment of the large intestine to confirm no angulin-2/ILDR1 mRNA was expressed (Fig. 4A). There was no detectable angulin-2/ILDR1 mRNA expression in each of the *Ildr1* knockout segments. Finally, to confirm the loss of angulin-2/ILDR1 protein in the colon, we performed immunofluorescence experiments staining with angulin-2/ILDR1 and another tight junction protein, occludin (Fig. 4B,C). Anti-angulin-2/ILDR1 antibody was raised against the cytoplasmic domain (aa259-537) of mouse angulin-2/ILDR1 protein^21^. There were no detectable angulin-2/ILDR1 immunofluorescence signals at tTJs and in the intracellular compartments in each *Ildr1* knockout segment (Fig. 4C), whereas bright punctate signals (indicated by arrow heads) for angulin-2/ILDR1 at tTJs were observed at the surface and in the crypts of wild-type mice (Fig. 4B). It is noteworthy that all *Ildr1* KO mice were deaf, which is another phenotype of loss of angulin-2 /ILDR1 in mice and humans^25,26^. Together, these results suggested that angulin-2/ILDR1 is not localized to tTJs of the colon in *Ildr1* KO mice.

**Table 1:** Metabolic cage experiment data.

Parameter	Wild-type	*Ildr1* KO	*Ildr1* KO (5 months)
Weight (before), g	23.3 ± 0.5	24.6 ± 1.0^n.s.^	27.9 ± 1.9^n.s.^
Water intake, g/day	6.5 ± 0.7	4.9 ± 0.3^n.s.^	5.2 ± 0.3^n.s.^
Food intake, g/day	4.0 ± 0.3	4.0 ± 0.3^n.s.^	3.6 ± 0.4^n.s.^
24 h urine, g/day	1.7 ± 0.2	1.6 ± 0.2^n.s.^	1.8 ± 0.3^n.s.^
24 h stool, g/day	2.1 ± 0.3	1.9 ± 0.3^n.s.^	1.5 ± 0.2^n.s.^
Fresh fecal water, %	57.7 ± 1.7	56.6 ± 1.3^n.s.^	53.3 ± 1.8^n.s.^

Mice aged 9–12 weeks were used in the first two columns. Data is presented as the mean of day 2 and day 3 averaged ± SEM, n = 6 (first two columns). Mice in the third column were 5 months old. Data is presented as the mean of day 2 and day 3 averaged ± SEM, n = 3.

*n.s.* not significant compared with wild-type mice.

### Colonic paracellular flux of ^22^Na^+^ and ^3^H-mannitol in *Ildr1* KO mice

Since ILDR1 is integral in tTJ of the large intestine, loss of angulin-2/ILDR1 could result in increased paracellular permeability and loss of intestinal barrier function. We first measured transepithelial electrical conductance (*G*_*t*_) in each segment of the large intestine (Table 2), however, there was no difference between *Ildr1* KO and WT mice, suggesting that the epithelial barrier function seems to be unaffected by the loss of angulin-2/ILDR1 (p = 0.20, p = 0.11 and p = 0.83, for proximal, middle and distal sections, respectively). Short circuit current (*I*_*sc*_) was also unchanged (Table 2), suggesting that transcellular transport was not changed in *Ildr1* KO mice (p = 0.56, p = 0.73 and p = 0.97, for proximal, middle and distal sections, respectively). We next directly assessed the paracellular pathway by measuring ^22^Na^+^ and ^3^H-mannitol flux, the properties of which are dependent on tight junctions. To investigate ionic permeability, the fluxes of ^22^Na^+^ were assessed in *Ildr1* KO mice (Table 2). Serosal–mucosal (S–M) flux was investigated as it is thought to occur mainly via the paracellular pathway^28^. As shown in Table 2, there is no difference in the S–M flux of ^22^Na^+^ in the proximal or middle section of the large intestine (p = 0.18 and p = 0.85, for proximal and middle sections, respectively). We next assessed leak pathways by using another paracellular maker, mannitol^29^, which is mainly transported by paracellular pathways. An increment of mannitol flux would indicate increased permeability of the epithelia and loss of barrier function. There is no difference in the S–M flux of ^3^H-mannitol (Table 2; p = 0.76 and p = 0.68, for proximal and middle sections, respectively). In addition, we measured the Na^+^ and K^+^ concentration of the luminal contents from the each intestinal segment (Table 2). There was no difference between *Ildr1* KO and WT mice.

**Table 2:** Barrier integrity and paracellular transport are maintained in the colon of *Ildr1* KO mice.

Intestinal section	Wild-type			*Ildr1* KO		
	Proximal	Middle	Distal	Proximal	Middle	Distal
Conductance (mS/cm^2^)	25.4 ± 1.6	21.7 ± 1.1	15.6 ± 1.8	28.7 ± 1.7^n.s.^	26.4 ± 2.3^n.s.^	16.7 ± 4.3^n.s.^
Isc (µA/cm^2^)	25.9 ± 10.8	14.3 ± 5.1	87.5 ± 29.9	18.0 ± 6.1^n.s.^	11.7 ± 4.8^n.s.^	89.2 ± 40.6^n.s.^
Na^+^ flux (µmol/cm^2^/h)	10.1 ± 0.2	10.5 ± 0.2	N.D.	10.8 ± 0.4^n.s.^	11.0 ± 2.3^n.s.^	N.D.
Mannitol flux (µmol/cm^2^/h)	0.30 ± 0.05	0.48 ± 0.03	N.D.	0.28 ± 0.03^n.s.^	0.45 ± 0.06^n.s.^	N.D.
Luminal Na^+^ (mM)	98.5 ± 9.3	56.4 ± 7.7	53.7 ± 14.4	116.5 ± 8.0^n.s.^	66.6 ± 15.7^n.s.^	89.9 ± 13.8^n.s.^
Luminal K^+^ (mM)	54.2 ± 10.7	71.8 ± 14.5	59.3 ± 5.4	39.2 ± 4.3^n.s.^	56.2 ± 5.1^n.s.^	64.6 ± 14.7^n.s.^

Intestinal sheets were prepared by removing the muscle layer and then mounted in Ussing chambers and the flux of ^22^Na^+^ and ^3^H-mannitol were measured, and electrical conductance calculated according Ohm’s Law for each large intestinal segment (n = 3–5). Na^+^ and K^+^ concentration of the luminal contents from each section of the large intestine were determined by Na^+^ and K^+^ electrodes (n = 3–5).

*n.s.* not significant compared with wild-type mice.

Taken together, it seems that paracellular transport is unaffected in *Ildr1* KO mice and there is no decrease in barrier integrity.

### Water transport appears to be unaffected in the *Ildr1* KO mouse colon

**Figure 5:**
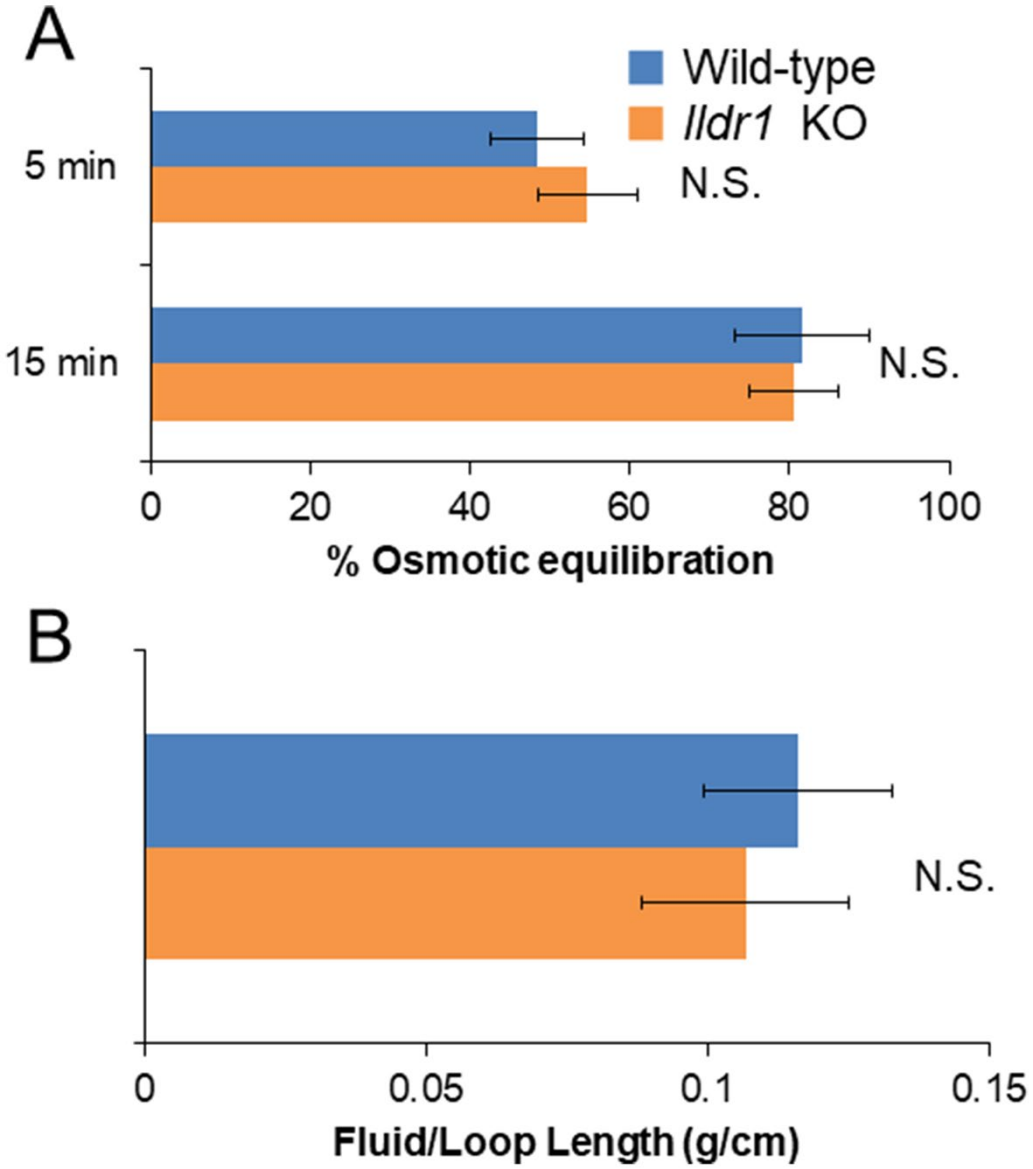
Closed loop analysis of hyperosmolar fluid secretion and cholera toxin-induced fluid secretion in *Ildr1* KO and WT mice. (**A**) % osmotic equilibration at 5 and 15 min in the descending colon of *Ildr1* KO and WT after injection with a hyperosmotic solution. n = 3–5. (**B**) Cholera toxin-induced swelling in ascending colonic loops in *Ildr1* KO and WT mice. Values are the average amount of fluid produced g/cm of intestine, n = 3–4. *N.S.* not significant compared with wild-type mice.

Naftalin proposed water absorption mechanisms in the colon^30^, however the route of water movement (transcellular or paracellular) and the driving force of water is not fully elucidated^31^. It was previously shown that angulin-2/ILDR1 is important for paracellular water transport in the kidney^23^ and since angulin-2/ILDR1 is the main tTJ protein in the large intestine^21^, in theory, paracellular water transport should be disrupted in the colon as well. To investigate water transport in the colon, we used a modified closed-loop procedure originally used to study the phenotype of aquaporin-8 knockout mice^10^. First, a hyperosmolar solution was injected into a loop in the middle colon and samples were withdrawn at 5 and 15 min. As shown in Fig. 5A, there was no difference in % osmotic equilibration in *Ildr1* KO and WT mice at 5 and 15 min after injection (p = 0.48 and p = 0.92, for 5 min and 15 min, respectively). In addition, the calculated water permeability coefficient (P_f_) did not differ between *Ildr1* KO and WT mice (0.0215 ± 0.0045 and 0.0183 ± 0.00224 cm/s at 5 min and 0.0104 ± 0.0019 and 0.0104 ± 0.0007 cm/s at 15 min, for KO and WT, respectively, n = 3–5, p = 0.55 and 0.97, for 5 min and 15 min, respectively). We then measured cholera toxin-induced fluid secretion by injecting cholera toxin into a closed loop in the ascending colon. Two hours after injection, the loop was removed and the length, weight, and amount of fluid was determined (Fig. 5B). In *Ildr1* KO mice, cholera toxin-induced swelling resulted in 0.107 ± 0.018 g of fluid/cm of intestine, which was not significantly different than that in WT mice (0.116 ± 0.017 g/cm, p = 0.72). Closed loop studies in *Ildr1* KO mice reveal that water transport is not interrupted in the large intestine.

### Another tricellular tight junction protein, angulin-1/LSR, replaces angulin-2/ILDR1 in the tricellular tight junctions of *Ildr1* KO mice

**Figure 6:**
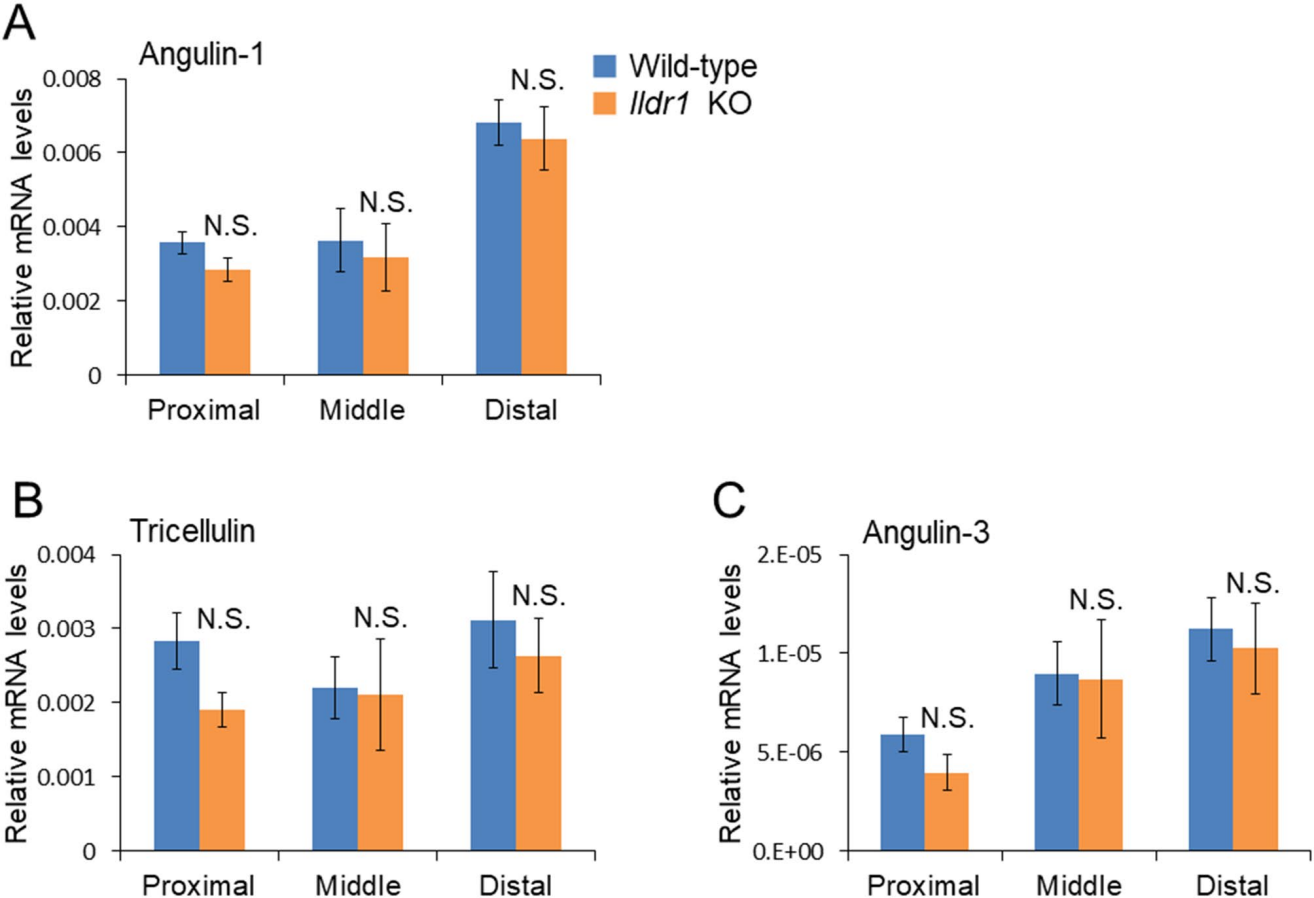
Relative mRNA levels of tTJ proteins in *Ildr1* KO and WT mice. Relative mRNA levels of (**A**) angulin-1/LSR, (**B**) tricellulin, and (**C**) angulin-3/ILDR2 in proximal, middle and distal sections of the large intestine in *Ildr1* KO and WT mice. n = 5. *N.S.* not significant compared with wild-type mice.

**Figure 7:**
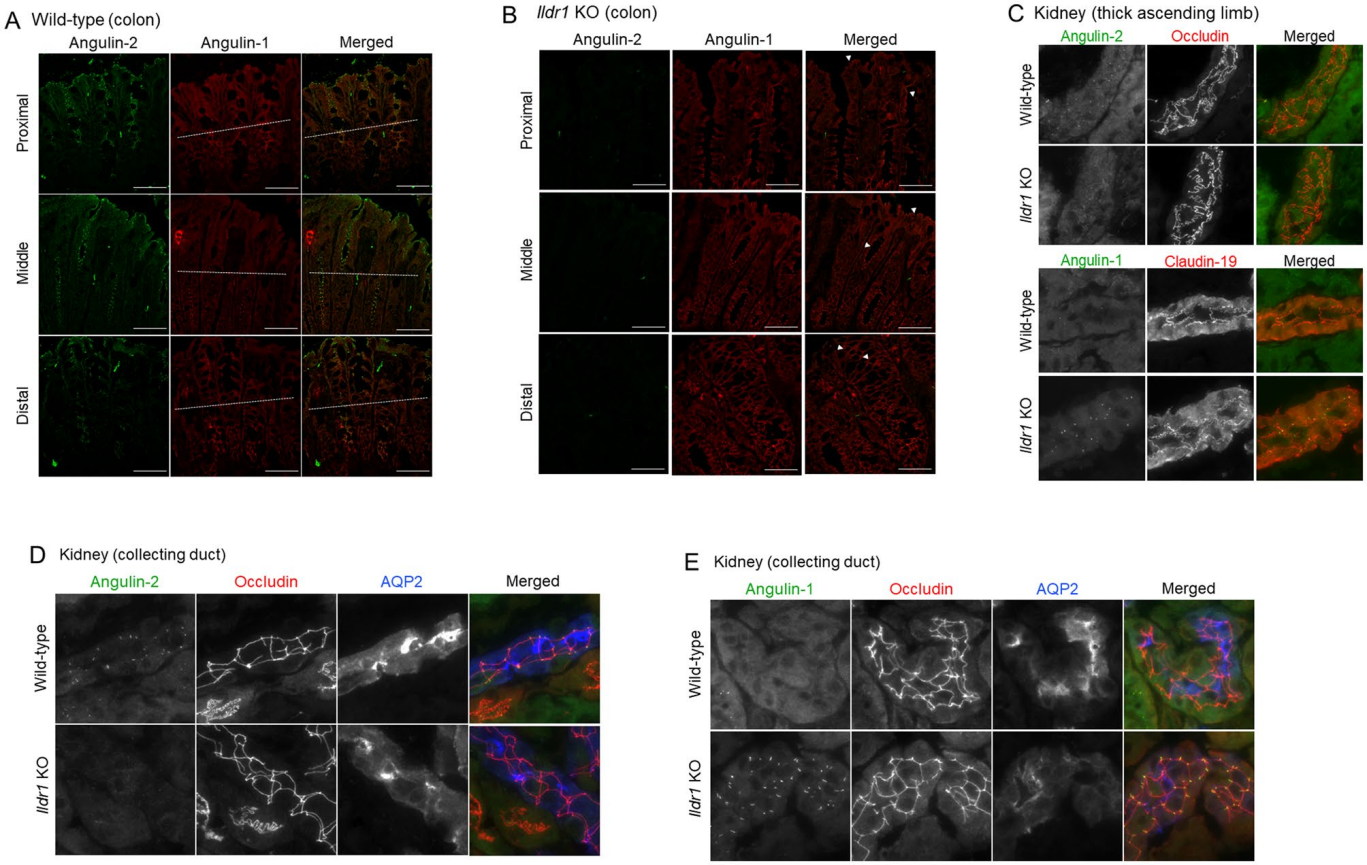
Immunofluorescence of angulin-2/ILDR1 and angulin-1/LSR in the large intestine and kidney of *Ildr1* KO and WT mice. Representative immunofluorescence images showing angulin-2/ILDR1 (green) and angulin-1/LSR (red) antibody staining in each section of the colon for (**A**) WT mice and (**B**) *Ildr1* KO mice. The dashed lines indicate the middle of the crypt. Scale bar 50 µm. Representative images showing angulin-2/ILDR1 and angulin-1/LSR in the (**C**) thick ascending limb (using occludin and claudin 19 as markers). Collecting duct (using occludin and AQP2 as markers) was stained with angulin-2/ILDR1 (**D**) and angulin-1/LSR (**E**) in the kidney of wild-type and *Ildr1* KO mice.

*Ildr1* KO mice do not seem to have a detectable phenotype aside from deafness, when comparing to WT controls, and previous experiments did not show alterations in colon transport and barrier function^24–26,32^. To investigate the reason for this, we examined the mRNA levels of the tTJ proteins, which are tricellulin, angulin-1 (LSR), angulin-2 (ILDR1), and angulin-3 (ILDR2)^33^. As shown in Fig. 6, there was no difference in the expression of angulin-1/LSR (Fig. 6A; p = 0.14, 0.72, and 0.69 for proximal, middle and distal sections, respectively), tricellulin (Fig. 6B; p = 0.07, 0.91, and 0.57 for proximal, middle and distal sections, respectively), and angulin-3/ILDR2 (Fig. 6C; p = 0.17, 0.93, and 0.74 for proximal, middle and distal sections, respectively) between *Ildr1* KO mice and WT mice. It seems that there is no upregulation of tTJ gene expression in response to angulin-2/ILDR1 deletion. We next assessed tissue localization of angulin-1/LSR by immunofluorescence (Fig. 7). As shown in Fig. 4B, angulin-2/ILDR1 is expressed throughout the surface and crypts of the colonic epithelium. This agrees with what has previously been shown for angulin-2/ILDR1 expression in WT mice^21^. In addition, it has been shown that angulin-1/LSR is co-expressed with angulin-2/ILDR1, but is restricted to the bottom third of the crypts, and is not specifically localized to tTJs^21^. It is speculated that angulin-1/LSR may compensate for decreased barrier function which would be induced by deletion of angulin-2/ILDR1. To test this hypothesis, we performed immunofluorescence experiments for angulin-1/LSR and angulin-2/ILDR1 in the large intestine (Fig. 7A,B). We observed co-localization of angulin-2/ILDR1 (green) and angulin-1/LSR (red) in the lower portion (below the white dashed line) of crypts in WT mice (Fig. 7A) as shown in previously^21^. In *Ildr1* KO mice, tissue localization of angulin-2/LSR was changed; it can be found in the TJ of the surface cells (indicated by arrowheads), which is not observed in wild-type mice, as well as the crypt cells (Fig. 7B). Perhaps this change in cellular localization can explain why there is no effect of angulin-2/ILDR1 KO on paracellular transport and barrier function in the large intestine. Similar changes may occur in the kidney of *Ildr1* KO mice, since we could not observe any water transport abnormalities as shown in Table 1 and Fig. 1. Therefore, we performed immunofluorescence experiments in the kidney of WT and *Ildr1* KO mice. In the thick ascending limb (TAL, Fig. 7C), which is impermeable to water and important for production of hypo-osmotic urine, angulin-2/ILDR1 was detected at TJ in WT mice (upper panel), while angulin-1/LSR was found to localize to the tTJ of KO but not WT mice (lower panel). Lastly, in the collecting duct (CD, Fig. 7D,E), which is permeable to water in the presence of antidiuretic hormone and important for production of hyperosmotic urine, angulin-2/ILDR1 was detected as punctate signals at tTJ in WT mice (Fig. 7D). However, angulin-1/LSR localized to the tTJ of KO mice unlike WT mice (Fig. 7E). In the TAL and the CD, angulin-1/LSR appears to compensate for the loss of angulin-2/ILDR1 by localizing to the tTJ, similar to what was observed in the large intestine.

**Figure 8:**
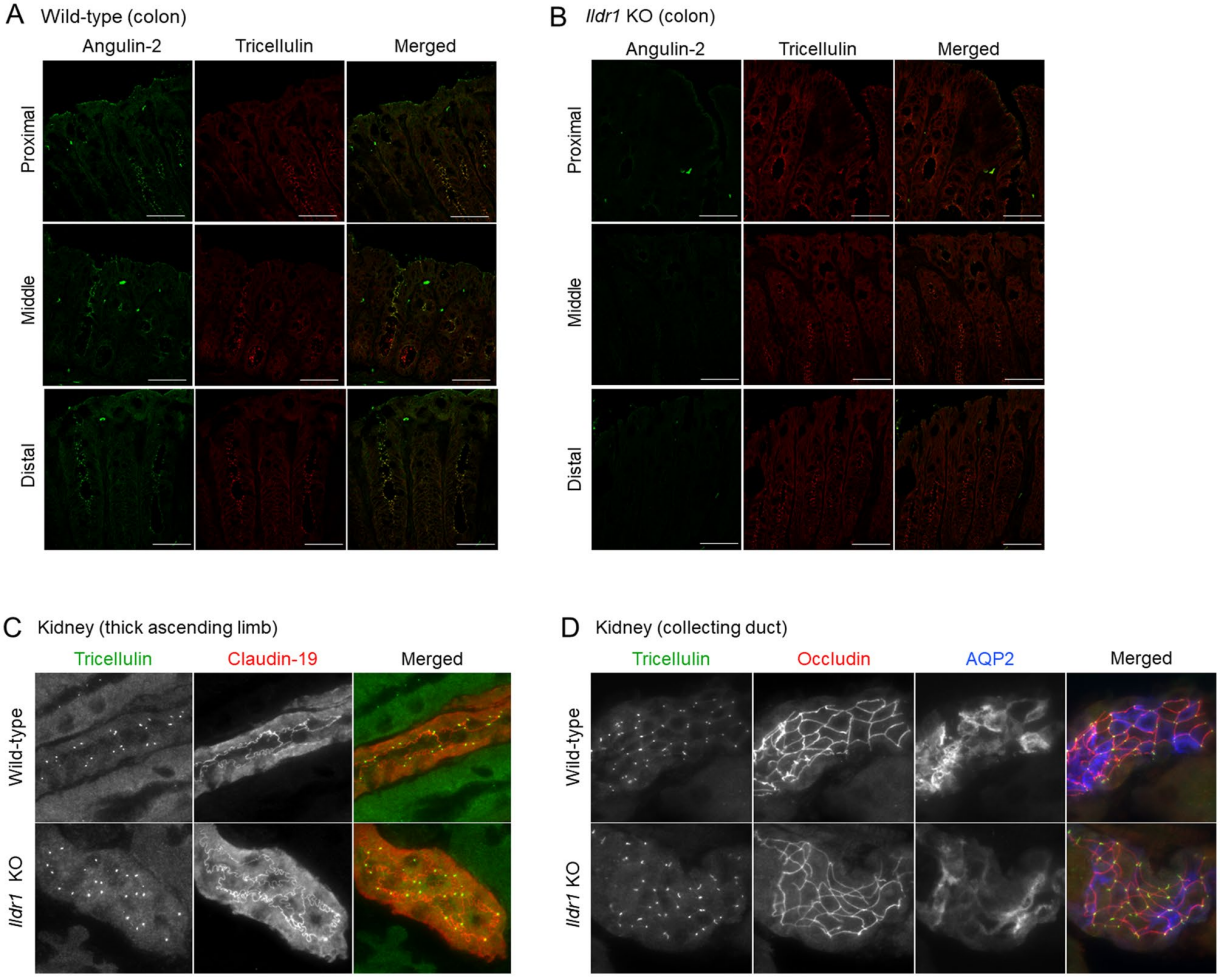
Immunofluorescence localization of tricellulin in *Ildr1* KO and WT mice large intestine and kidney. Representative immunofluorescence images showing angulin-2/ILDR1 (green) and tricellulin (red) antibody staining in each section of the colon for (**A**) WT mice and (**B**) *Ildr1* KO mice. Scale bar 50 µm. Representative images showing tricellulin in the (**C**) thick ascending limb (using occludin and claudin 19 as markers) and (**D**) collecting duct (using occludin and AQP2 as markers) in the kidney of WT and *Ildr1* KO mice.

We also assessed the tTJ protein, tricellulin. Tricellulin has been recently shown to be involved in water transport in MDCK c7 cells^22^, so we checked to see whether tricellulin localization is also affected by *Ildr1* knockout in the kidney and the large intestine. In each section of the large intestine and the TAL and CD in the kidney of WT and *Ildr1* KO mice, there was no difference in the localization of tricellulin (Fig. 8).

## Discussion

*ILDR1* mutation in humans leads to the autosomal recessive deafness syndrome, DFNB42^34^. This has been shown in mice where angulin-2/ILDR1 loss leads to gradual mislocalization of tricellulin in inner ear sensory epithelial cells causing degeneration of the hair cell structure during early postnatal development^25^. Angulin-2/ILDR1 was also shown to be important in the murine kidney, as *Ildr1* KO mice are reported to be unable to concentrate urine^23^. As such, the first goal of this research was to establish the intestinal phenotype of *Ildr1* KO mice.

To assess the physiological parameters in *Ildr1* KO mice, the mice were put into metabolic cages, however, there was no difference observed between *Ildr1* KO and WT mice (Table 1). Previously published data showed that *Ildr1* KO mice have polydipsia (35 mL water/day) and polyuria (8 mL urine/day)^23^. In this study, *Ildr1* KO mice consumed 5 mL water/day, and 24 h urine output was 2 mL/day (Table 1). The discrepancy between previous results^23^ and our results is not known. Other studies published about *Ildr1* KO mice did not focus on kidney function, however there was no mention of polyuria or polydipsia^24–26^. In addition, to our knowledge, in patients with genetic mutation of *ILDR1*, there have been no reports of altered intestinal or renal water transport. Mutation of the *ILDR1* gene is associated with autosomal recessive non-syndromic hearing loss, which means patients have no other major symptoms other than hearing loss clinically^34^. In addition, no anomalies were detected in blood chemistry or other physiological properties in human DFNB42 patients^34^.

The bodyweight of *Ildr1* KO mice was tracked from weaning (4 weeks) until about 21 weeks of age (Fig. 3) and there was no difference in growth of *Ildr1* KO and WT mice. This result is not consistent with Gong’s study^23^, which showed growth retardation in *Ildr1* KO mice. Given that growth curves seem to differ among *Ildr1* KO mouse models, it is possible that mouse genetic background and genetic manipulation are contributing factors to the differences observed. Both mouse models result in deafness, however, suggesting that they are not so different from each other. Deafness due to degeneration of the hair cell structure gradually developed resulting in deafness at about 30 days after birth^25^, which might indicate that the phenotype of *Ildr1* KO mice may be a late-onset phenotype. However, this explanation is unlikely on the basis of the observation that there were no discernable differences between older 5 month old *Ildr1* KO mice and young WT mice (Table 1, third column).

Tricellular TJ are theorized to play an important role in paracellular transport, however a mechanism has not yet been elucidated. At tTJ, the sealing elements from the three cells come together to form the central sealing element which forms a pore of about ~ 10 nm diameter^20^. Because of its size, the central sealing element of tTJ is thought to be a good candidate for water transport and transport of larger solutes. If this idea is correct, disrupting the tTJ structure should result in “leaky” epithelium, due to the loss of the sealing elements. It has been demonstrated that angulin family members are important for barrier function in angulin-1/LSR knock-down EpH4 cells^21^. *Ildr1* KO mice did not present with diarrhea or watery stools, which would indicate impaired transport in the colon. Analysis of fresh stool from *Ildr1* KO mice revealed no changes in water content or Na^+^ and K^+^ concentration (Table 1). In addition, the water content of the luminal contents was also not changed (Proximal: 75.4 ± 4.2 vs. 72.3 ± 1.0%, n = 5 for *Ildr1* KO and WT, respectively, p = 0.50; Middle: 57.4 ± 2.2 vs. 58.9 ± 1.5%, n = 5 for *Ildr1* KO and WT, respectively, p = 0.58; Distal: 51.3 ± 0.3 vs. 57.4 ± 2.9%, n = 3 for *Ildr1*1 KO and WT, respectively, p = 0.17). To evaluate the barrier function of *Ildr1* KO mice, the fluxes of ^22^Na^+^ and ^3^H-mannitol were measured. However, there were no discernable differences between *Ildr1* KO and WT mice (Table 2). It seems that the barrier function of the large intestine of *Ildr1* KO mice remains intact. This conclusion raises the question how tTJ are fortified in the absence of angulin-2/ILDR1 protein. We believe that another angulin family member, angulin-1/LSR, compensates for the loss of angulin-2/ILDR1 at tTJ.

Angulin-1/LSR is coexpressed with angulin-2/ILDR1 in the large intestine and the kidney^21^. Alterations to angulin-1/LSR expression can have an effect on barrier function. For example, knockdown of angulin-1/LSR in an epithelial cell line resulted in diminished barrier function and tTJ formation was affected^35^. We found that angulin-1/LSR mRNA levels did not change (Fig. 6), however, in each section of the large intestine and in the TAL and CD of the kidney, angulin-1/LSR localization is changed (Fig. 7). In the large intestine of WT mice, angulin-1/LSR is localized in the bottom half of the crypts, while angulin-2/ILDR1 is localized throughout the crypt and the surface (Fig. 7A). However in angulin-2/ILDR1 KO mice, the localization of angulin-1/LSR resembles that of wild-type angulin-2/ILDR1 expression (Fig. 7B). We propose that angulin-1/LSR localizes to the tTJ in the absence of angulin-2/ILDR1 in the colon and rescues the phenotype of *Ildr1* KO mice. This compensation mechanism is not completely efficient, because while water transport did not seem to be impaired under normal conditions, after 24 h water restriction, *Ildr1* KO mice were unable to concentrate urine to the same degree as control mice (Fig. 2). This shows that although angulin-1/LSR can compensate for angulin-2/ILDR1, the roles of the two proteins are slightly different (which is reflected in their tissue distribution, a detailed list of which was first established by Higashi et al.^21^) and angulin-1/LSR is not 100% effective at maintaining the urine concentration mechanism.

The mechanism of water absorption in the colon is largely unknown. The paracellular pathway may be involved in water transport in the colon. A recent study with MDCK cells revealed that tricellulin knockdown leads to increased water flux^22^. In addition, angulins were found to recruit tricellulin to tTJ and both an angulin and tricellulin are required for the full barrier function in MDCK cells^21^. We examined the mRNA expression levels of tricellulin (Fig. 7) as well as cellular localization by immunofluorescence in the kidney and the large intestine and kidney (Fig. 8), but there was no change in tricellulin observed in *Ildr1* KO mice, which is consistent with the lack of change in water flux observed in *Ildr1* KO mice. Although the intestinal phenotype was examined in tricellulin KO mice^36^, tricellulin is not likely be involved in water transport, since tricellulin mutation in human leads to non-syndromic deafness with no other clinical manifestations^37^. However, further experiments are needed to clarify the possibility of tricellulin involvement in water transport in the intestine.

In summary, we concluded that *Ildr1* KO mice have no detectable water transport abnormalities. In addition, we found that in the colon and the kidney of *Ildr1* KO mice, another tTJ protein, angulin-1/LSR, changes its expression pattern and this change in tissue localization of angulin-1/LSR compensates for the loss of angulin-2/ILDR1 and maintains the barrier function of the epithelia.

## Materials and methods

### Animals

*Ildr1* KO mice (Ildr1^*tm1(KOMP)Wtsi*^) on a C57BL/6J background were originally obtained from the Wellcome Trust Sanger Institute Mouse Genetics Project (Sanger MGP) as described previously^26^.

All mice were bred and maintained in the University of Shizuoka animal care facility. ILDR1 knockout was confirmed by isolation of genomic DNA from tail snips using GenElute Mammalian Genomic DNA Miniprep Kit (Sigma-Aldrich, St. Louis, MO). PCR Genotyping was performed using specific primers pairs (Common, 5′-CGAAGGAATCTTTCCAAATTGAGGC-3′; WT, 5′-ATCCATAGACCAAGTTCCAGGGAAG-3′; KO, 5′-AGGAACTTCGGAATAGGAACTTCGG-3′). All experiments conducted on animals were approved by the Animal Care and Use Committee of the University of Shizuoka (permit #195221 and #655-2203) and were conducted in accordance with the guidelines set out for use of animals by the University of Shizuoka. Sex-matched wild type mice (where possible, siblings) were used as controls. Mice were fed a standard pellet diet (CE-2, Clea, Tokyo, Japan) and were given ad libitum access to food and water and housed in a temperature and humidity-controlled environment with a 12 h light/dark cycle.

### Metabolic cage

Mice were kept individually in metabolic cages (Tecniplast, Buguggiate, Italy) for 3 days. They were fed a powder diet (CE-2, Clea, Tokyo, Japan) and had free access to both food and water. Food and water intake was measured daily at 11.00 h, and urine and stool were collected in pre-weighed tubes for analysis. The fecal pellets and urine were stored in test tubes at − 30 °C before analysis. Body weight was measured each day, and fresh stool was collected daily at 11.00 h in pre-weighed tubes by stimulating the anal sphincter with a cotton swab.

### Urine, stool, and intestinal contents analysis

Urine and stool samples collected from the metabolic cage experiment were weighed and stool samples were dried for 24 h in a drying oven at 80 °C. Urine Na^+^ and K^+^ were analyzed by pipetting 50 µL undiluted urine onto a Na^+^ or K^+^ electrode (LAQUAtwin; Horiba, Kyoto, Japan). Fresh stool (1–3 pieces) collected directly from the mice was weighed, dried for 24 h at 80 °C in a drying oven, and weighed again. The amount of water in the stool was calculated by subtracting the dried weight from the wet weight. The dried stool was then dissolved in 500 µL deionized water. To dissolve it completely into water, the samples were heated in a heat block for 1–2 h at 80 °C, and then ground with spatula and mixed by vortexing. Samples were then centrifuged for 10 min at 12,000 rpm and the supernatant was collected for Na^+^ and K^+^ analysis. Fresh urine was also isolated directly from the bladder under anaesthesia with a mixture of three drugs by intraperitoneal injection (10 µL/g body weight) consisting of medetomidine (30 µg/mL; Nippon Zenyaku Kogyo, Fukushima, Japan), midazolam (0.4 mg/mL; Teva Pharma, Nagoya, Japan), and butorphanol (0.5 mg/mL; Meiji Seika, Tokyo, Japan). Urine samples were analyzed for osmolality (Osmostat OM-6040, Arkray Inc., Kyoto, Japan), and Na^+^ and K^+^ concentrations.

### Flux and electrical conductance measurements

Measurement of electrical parameters and transepithelial flux under short-circuit conditions were performed as previously described with modifications^38^. Briefly, mice were anaesthetized with a mixture of three drugs. Mice were opened with a midline incision to the abdomen. The large intestine was excised and split into thirds equal in length (proximal, middle, and distal) and each section was opened longitudinally. Intestinal sheets were rinsed well in ice cold Ringer’s solution (containing in mM: 119 NaCl, 21 NaHCO_3_, 2.4 K_2_HPO_4_, 0.6 KH_2_PO_4_, 1.2 CaCl_2_, 1.2 MgCl_2_, 10 d-glucose, and 10 µM indomethacin) which was gassed with 95% O_2_–5% CO_2_ (pH 7.4). The muscle layer was removed under a stereo-microscope and intestinal sheets were then mounted in Ussing chambers with a window area of 0.2 cm^2^ and bathed in 5 mL ringer solution kept at 37 °C by a water jacket. As previously described by Ishizuka et al.^39^, intestinal tissue was continuously short-circuited using a voltage-clamping amplifier (CEZ9100; Nihon Kohden, Tokyo, Japan). When current flowed from the mucosal side (M side) to the serosal side (S side), the short-circuit current (*I*_*sc*_) was interpreted as positive. Transepithelial tissue conductance (*G*_*t*_) was calculated from the change in current after ± 10 mV voltage pulses, according to Ohm’s Law. The unidirectional transepithelial radio-active isotope flux of ^22^Na^+^ and ^3^H-mannitol in the serosal-to-mucosal direction was measured as described previously^39^. Briefly, five samples (0.5 mL each) were taken from the unlabeled side every 30 min and immediately replaced with an equal volume of unlabeled solution. Samples containing ^22^Na^+^ or ^3^H-mannitol were counted in a liquid scintillation counter (LSC-7500; Aloka, Tokyo, Japan).

### Intestinal fluid transport measurement in vivo

Animals were anaesthetized with isoflurane (Wako Pure Chemical Industries, Osaka, Japan) administered by an animal anesthetizer (TK-7; Bio Machinery, Funabashi, Japan) at 1.5 L/min and 2–3% isoflurane. Procedures were performed on a warming plate to maintain animal’s body temperature for the duration of the experiment. The abdomen was opened by a midline incision and the large intestine was exposed. 1–1.5 cm segments of the ascending or descending colon were tied off using sutures to create closed loops^10^. For the hyperosmolar induced fluid secretion assay, 100–150 µL of hyperosmotic solution (0.5% blue dextran in PBS containing 300 mM mannitol) was injected into the loop. Five minutes and 15 min after injection, samples were withdrawn and osmolality was measured using an osmometer. For cholera toxin-induced fluid secretion, cholera toxin (4 µg/0.1 mL PBS) was injected into the colonic loop. The animal abdomen was closed, and after 2 h, the colonic loop was excised, and loop weight, length, and luminal fluid content were determined.

### Real time quantitative PCR

Real time quantitative PCR experiments were performed as previously described^38^. Briefly, total RNA of the mucosal tissue was isolated and eluted using NucleoSpin RNA columns (MACHERY-NAGEL, Düren, Germany) according to the manufacturer’s instructions. RNA was reversed transcribed to cDNA templates which were mixed with primers and SYBR Ex Taq II. mRNA levels were normalized to β-actin and calculated using the ΔΔCt method. Primers and sequences used for PCR are shown in Table 3.

**Table 3:** List of primers used for quantitative real-time PCR.

	Forward primer (5′ → 3′)	Reverse primer (5′ → 3′)
β-Actin	5′-CATCCGTAAAGACCTCTATGCCAAC-3ʹ	5′-ATGGAGCCACCGATCCACA-3ʹ
Angulin-1 (LSR)	5′-AGACAAGTGCTGTTGCCCTGA-3ʹ	5′-CATAGATGCTTGGCACACCTGA-3ʹ
Angulin-2 (ILDR1)	5′-ATCACCATCCAGAACCGAG-3ʹ	5′-CACCAGCATACACCAATCAG-3ʹ
Angulin-3 (ILDR2)	5′-TCTGGATGGGAAGTTGGGGA-3ʹ	5′-TGGGAGGACGTGGAAAAGTG-3ʹ
Tricellulin	5′-GCAGGCTCCCACATCATTCTG-3ʹ	5′-TTGAGGTAATCGCAACGCTCC-3ʹ

### Immunofluorescence

The large intestine was excised and divided into three segments as in the flux experiments. Each segment was opened and rinsed with ice cold PBS. The tissue segment was coated with Tissue-Tek O.C.T. compound (Sakura Finetek, Tokyo, Japan), and embedded into a mold containing O.C.T. compound and frozen at − 80 °C. The kidneys were also excised and cut into 2 mm^3^ pieces and embedded into a O.C.T. compound and frozen at − 80 °C. Specimen blocks were stored at − 80 °C until the day of thin sectioning. Specimen blocks were sectioned into 5 µm slices using a Cryostat (CM3050 S; Leica Biosystems, Nussloch, Germany) and put on coverslips. Sections were dried under a fan for 30 min, and then incubated in 95% ethanol on ice for 30 min. Coverslips were then bathed in acetone for one minute and rinsed three times in PBS. The tissue was pre-blocked with 5% skim milk powder in 0.1% Triton X-100 in PBS (0.1% PBST) for 30 min. The coverslips were incubated with primary antibodies for either angulin-2 (ILDR1), angulin-1 (LSR) or tricellulin^21^ for 30 min. After washing in PBS, coverslips were incubated with secondary antibodies (1:1,000 dilution) conjugated with Alexa Fluor 488 (Abcam, Cambridge, UK) or Alexa Fluor 546 (Invitrogen, Carlsbad, CA). After washing, the coverslips were mounted onto glass slides with mounting medium (Fluoromount-G; SBA Southern Biotechnology Associates, Inc., Birmingham, AL). Tissue was visualized using a laser scanning microscope (LSM700; Zeiss, Oberkochen, Germany).

### Statistical analyses

Experimental values are given as the means ± SE of the indicated number of the determinations. Comparisons between two groups were made with unpaired Student’s *t* test. In all instances, p < 0.05 was considered to be statistically significant.

## Data Availability

Raw data is available upon request to the corresponding author.
